# Letter to the editor regarding: ‘association of CXCL16 in aseptic loosening following total hip arthroplasty and its effect on bone metabolism: a case-control study’

**DOI:** 10.1080/07853890.2025.2610547

**Published:** 2025-12-31

**Authors:** Sheng Zheng, Jinjin Luo, Yong Zhu

**Affiliations:** Department of Orthopedics, Yuyao Hospital of Traditional Chinese Medicine, Yuyao, China

We commend Zhu et al. for establishing a robust correlation between CXCL16 overexpression and RANKL/OPG imbalance in aseptic loosening (AL) [1]. Their work provides valuable mechanistic insights into macrophage-driven osteoclast activation. However, three critical gaps limit clinical translation and warrant scholarly dialogue.

First, the absence of non-invasive biomarker validation restricts CXCL16’s utility in early AL detection. While tissue-based correlations are compelling, circulating CXCL16 levels remain unquantified. Serum/plasma quantification *via* longitudinal cohort studies—correlated with PET-CT osteolysis signatures—is essential to establish diagnostic thresholds and dynamic monitoring protocols. Integrating multi-omics (e.g. CXCL16 + miR-451a synergy in osteolysis regulation) could enhance specificity beyond inflammatory confounders.

Second, prosthetic material opportunities were underdeveloped. The observed lower CXCL16 expression with ceramic couplings suggests implant surface engineering could preemptively modulate macrophage polarization. Nano-textured interfaces promoting anti-inflammatory M2c phenotypes merit exploration to disrupt CXCL16-RANKL crosstalk. Concurrently, repurposing anti-CXCL16 monoclonal antibodies for high-risk patients might extend prosthesis longevity—a hypothesis testable through adaptive trial designs.

Third, socioeconomic dimensions were overlooked. Aseptic loosening is the most commonly reported reason for revision surgery, imposing a substantial financial burden on patients [2]. Embedding cost-effectiveness analyses within biomarker validation studies (e.g. CXCL16 screening vs. revision expenses) would align discovery with value-based orthopedics. Federated analysis of joint registries could further reveal disparities in AL burden related to demographics or access barriers, guiding equitable prevention strategies.

Spatiotemporal dynamics of CXCL16 also remain unaddressed. Spatial transcriptomics mapping peri-implant osteolytic niches and graph neural networks modeling wear particle kinetics could unveil CXCL16’s role in biomechanical failure cascades. Such AI-driven approaches might predict individualized AL trajectories beyond static correlations.

In summary, elevating CXCL16 from a biomarker candidate to a clinical tool requires validating circulatory assays, pioneering immunomodulatory implants, and integrating health economics. This trifecta could transition AL management from reactive revision to precision prevention, ultimately enhancing global joint arthroplasty sustainability.

## Data Availability

Data sharing is not applicable to this article as no data were created or analysed in this study.
